# Phytochemical screening and antioxidant parameters data in prostatic rats fed with *Laportea aestuans* leaves

**DOI:** 10.1016/j.dib.2018.08.020

**Published:** 2018-08-21

**Authors:** Omotosho Omolola E., Oladipupo O. Ogunlade, Abiodun O. Salako

**Affiliations:** Department of Biochemistry, Covenant University, P.M.B. 1023, Km. 10, Idiroko road, Canaan land, Ota, Ogun State, Nigeria

## Abstract

Several plants have been used in ancient times as medicines to treat, manage and prevent many diseases in various traditional settings throughout the world. The effect of administration of hydro-ethanolic extract of *Laportea aestuans* (La) leaves at different doses in Wistar rats induced with benign prostatic hyperplasia (BPH) using antioxidant parameters and phytochemical screening data was obtained. Thirty (30) animals were randomly divided into six (6) groups (A-F) of five (5) animals each. BPH was induced in the animals by daily subcutaneous injection of testosterone propionate (TP) (3 mg/kg) in olive oil and administration of treatments for four (4) weeks were done concurrently. Group A received olive oil alone subcutaneously, group B was induced with BPH alone, groups C-E were induced with BPH but received different doses of La at 100, 200 and 400 mg/kg. Lastly, group F was induced with BPH but treated with finasteride (5 mg/kg) which serves as the positive control group. Phytochemical screening data of saponins, flavonoids (0.5010 ± 0.0009 mg/ml), alkaloids (0.528 mg/ml), phenols (0.6195 ± 0.0015 mg/ml), tannins (0.5410 ± 0.0013 mg/ml) and steroids (1.6230 ± 0.0210 mg/ml) in hydro-ethanolic extract of La. Antioxidant parameters such as superoxide dismutase, catalase and reduced glutathione data were alsoµ gotten at 400 mg/kg La (48.1 ± 4.17U/mg protein), (29.43 ± 1.38U/mg protein) and (30.60 ± 2.05 µg/ml) respectively when compared to the BPH group (35.5 ± 0.97U/mg protein), (11.36 ± 2.39U/mg protein) and (15.60 ± 1.14 µg/ml).

**Specifications Table**

**Table:** 

Subject area	Biochemistry
More specific subject area	Pharmacology and medicinal plant research
Type of data	Tables
How data was acquired	mean ± SEM, spectrophotometer, weighing balance, rotary evaporator.
Data format	Raw, Analysed
Experimental factors	*Laportea aestuans* was macerated, soaked in 80% ethanol and extracted using rotary evaporator. Animals (Wistar rat) gotten were acclimatized, induced with benign prostatic hyperplasia and treated with *Laportea aestuans* and finasteride.
Experimental features	Quantitative and qualitative phytochemical screening was assayed on plant sample, antioxidant parameters such as catalase, superoxide dismutase and reduced glutathione was assayed on animal plasma of different groups.
Data source location	Ota, Ogun State, Nigeria
Data accessibility	Data available within the article

**Value of the data**

• The data presented gives the quantitative assessment of certain phytochemicals responsible for the beneficial property of *Laportea aestuans* being used as an anti-hyperplastic agent.
• The data given may correlate with the results obtained using same plant in other regions.
• Data given can influence the development of a new drug against benign prostatic hyperplasia without extravagant side unlike finasteride.
• The data obtained from antioxidant assays may correlate with data obtained in other regions with a different plant or similar plant.

## 1 Experimental design, materials and methods

### 1.1 Plant material

Fresh *Laportea aestuans* were collected within Sango-Ota, Ogun State, Nigeria and was identified (UIH-22638) at the Botany Department of University of Ibadan, Ibadan, Oyo State, Nigeria. The leaves were picked from the branch, air dried and ground to powdered form before use. The pulverized leaves were weighed (600 g), soaked for 3 days in 80% ethanol, sieved and extracted (32 g) using a rotary evaporator.

### 1.2 Phytochemical screening

Qualitative and quantitative phytochemical screening was carried out according to the method described by [7], [8] respectively. Quantitative phytochemical screening of total phenol was carried out at a wavelength of 765 nm, tannins at 640 nm, steroid at 640 nm and flavonoid at 415 nm.

### 1.3 Animals

A total of thirty male *Wistar* rats (*Rattus norvegicus*) weighing 170 g to 230 g were purchased from the Federal University of Agriculture, Abeokuta, Nigeria. The rats were housed in the Animal house of Covenant University at room temperature and alternating light cycle 12 h light and dark cycle. All experimental procedures were carried out in compliance with Guidelines for the Care and Use of Laboratory Animals prescribed and approved by Covenant University Health Research Ethics Committee (CHREC/016/2018). After one week of acclimatization, the rats were randomly divided into six (6) groups of five (5) rats each.

### 1.4 Animal treatment

Group A: subcutaneous injection of olive oil. (Normal control group)
Group B: no treatment. 3 mg/kg body weight TP (BPH control group)
Group C: 100 mg/kg body weight *Laportea aestuans* orally and 3 mg/kg body weight TP
Group D: 200 mg/kg body weight *Laportea aestuans* orally and 3 mg/kg body weight TP
Group E: 400 mg/kg body weight *Laportea aestuans* orally and 3 mg/kg body weight TP
Group F: 5 mg/kg body weight finasteride and 3 mg/kg body weight TP.

Finasteride was used as the standard drug against BPH and as a positive control. The subcutaneous injection of TP, finasteride and oral administration of the extracts by oral gavage were done daily concurrently [6]. The rats had unlimited access to food and water throughout the phase of the experiment which lasted for 4 weeks. The body weight was measured weekly. The application volumes were 5 mg/ml/kg for oral administration of finasteride, 100 mg/ml/kg, 200 mg/ml/kg, 400 mg/ml/kg of *Laportea aestuans* at varying doses and 3 mg/ml/kg for subcutaneous injection of testosterone propionate. After the final treatment, the rats were fasted overnight and anesthetized using diethylether (DEE).

### 1.5 Collection of blood and prostate samples

Blood was collected by cardiac puncture into heparinized bottles for antioxidant assays. The blood was spun at 3500 rpm for 15 min to obtain the plasma [2], [3] and then stored at −4 °C for further analysis.

### 1.6 Determination of antioxidant status

Antioxidant assays carried out were superoxide dismutase (SOD), catalase (CAT) and glutathione (GSH) estimation. Also, the protein concentration was determined. Protein determination was determined according to [4] at a wavelength of 660 nm. SOD was determined by the method of [5] at 480 nm. CAT activity was determined according to the method of Claiborne, 1985 at 240 nm. The level of GSH was estimated according to the method of [1] at a wavelength of 412 nm ( Table 1, Table 2).

**Table 1:** If Qualitative phytochemicals in Laportea aestuans.

Phytochemical	Presence/absence
Saponins	+ve
Alkaloid	+ve
Flavonoid	+ve
Terpenoids	-ve
Phenols	+ve
Tannins	+ve
Glycosides	-ve
Steroid	+ve

**Table 2:** Quantitative phytochemicals present in *Laportea aestuans*.

Phytochemical	Concentration (mg/ml)
Steroid	1.6230 ± 0.0210
Tannin	0.5410 ± 0.0013
Phenol	0.6195 ± 0.0015
Flavonoid	0.5010 ± 0.0009
Alkaloid	0.5280

Values are expressed as mean±SEM (standard error of mean).

## 2 Data

### 2.1 Qualitative phytochemical screening

## 3 Quantitative Phytochemical Screening

## 4 Evaluation of oxidative stress biomarkers in blood plasma of experimental animals

 See  Table 3, Table 4, Table 5.

**Table 3:** Catalase (CAT) data in rats induced with benign prostatic hyperplasia and treated with *Laportea aestuans*.

Groups	Concentration (U/mg protein)
A	34.17 ± 1.36
B	11.36 ± 2.39
C	22.33 ± 1.94
D	25.66 ± 2.22
E	29.43 ± 1.38
F	30.30 ± 0.64

**Table 4:** Superoxide dismutase (SOD) data in rats induced with benign prostatic hyperplasia and treated with *Laportea aestuans*.

Groups	Concentration (U/mg protein)
A	55.10 ± 1.63
B	35.50 ± 0.98
C	42.10 ± 0.89
D	47.00 ± 1.72
E	48.10 ± 4.17
F	54.40 ± 1.49

**Table 5:** Reduced glutathione (GSH) data in rats induced with benign prostatic hyperplasia and treated with *Laportea aestuans*.

Groups	Concentration (µg/ml)
A	31.60 ± 3.82
B	15.60 ± 1.14
C	22.80 ± 0.08
D	27.50 ± 0.49
E	30.60 ± 2.05
F	26.50 ± 0.40

Values are expressed as mean ± SEM (standard error of mean).

## 5 Statistical analysis

Statistical analysis was carried out using GraphPad Prism (version 7.04 for Microsoft Windows 10) significance by one way analysis of variance (ANOVA), then Fischer׳s Test for post hoc comparison. Data were expressed as mean ± SEM (standard error of mean) of five replicates. Microsoft Excel 2013 was also used in plotting standard curves. Values of *p*< 0.05 were considered statistically significant.
